# Fungal Cell Gigantism during Mammalian Infection

**DOI:** 10.1371/journal.ppat.1000945

**Published:** 2010-06-17

**Authors:** Oscar Zaragoza, Rocío García-Rodas, Joshua D. Nosanchuk, Manuel Cuenca-Estrella, Juan Luis Rodríguez-Tudela, Arturo Casadevall

**Affiliations:** 1 Servicio de Micología, Centro Nacional de Microbiología, Instituto de Salud Carlos III, Majadahonda, Madrid, Spain; 2 Department of Microbiology and Immunology, Albert Einstein College of Medicine, Bronx, New York, United States of America; 3 Department of Medicine, Albert Einstein College of Medicine, Bronx, New York, United States of America; Carnegie Mellon University, United States of America

## Abstract

The interaction between fungal pathogens with the host frequently results in morphological changes, such as hyphae formation. The encapsulated pathogenic fungus *Cryptococcus neoformans* is not considered a dimorphic fungus, and is predominantly found in host tissues as round yeast cells. However, there is a specific morphological change associated with cryptococcal infection that involves an increase in capsule volume. We now report another morphological change whereby gigantic cells are formed in tissue. The paper reports the phenotypic characterization of giant cells isolated from infected mice and the cellular changes associated with giant cell formation. *C. neoformans* infection in mice resulted in the appearance of giant cells with cell bodies up to 30 µm in diameter and capsules resistant to stripping with γ-radiation and organic solvents. The proportion of giant cells ranged from 10 to 80% of the total lung fungal burden, depending on infection time, individual mice, and correlated with the type of immune response. When placed on agar, giant cells budded to produce small daughter cells that traversed the capsule of the mother cell at the speed of 20–50 m/h. Giant cells with dimensions that approximated those in vivo were observed in vitro after prolonged culture in minimal media, and were the oldest in the culture, suggesting that giant cell formation is an aging-dependent phenomenon. Giant cells recovered from mice displayed polyploidy, suggesting a mechanism by which gigantism results from cell cycle progression without cell fission. Giant cell formation was dependent on cAMP, but not on Ras1. Real-time imaging showed that giant cells were engaged, but not engulfed by phagocytic cells. We describe a remarkable new strategy for *C. neoformans* to evade the immune response by enlarging cell size, and suggest that gigantism results from replication without fission, a phenomenon that may also occur with other fungal pathogens.

## Author Summary

In this article we describe the formation of giant cells by the human fungal pathogen *Cryptococcus neoformans* during infection, involving an approximately 900-fold increase in volume compared to that of yeast cells grown in vitro. This switch to gigantism is a dramatic transition that is posited to have important consequences during infection. The paper reports the phenotypic characterization of these cells and the relationship between giant cell formation and polyploidy which suggests that gigantism is achieved by continued cell growth and DNA replication without fission. During infection, we observed an inverse correlation between the proportion of giant cells in the lung of infected mice and the inflammatory response elicited by the animals. In conclusion, our results indicate that during infection, *C. neoformans* forms giant cells, which might be implicated in fungal survival in the host during long time periods, especially during chronic and asymptomatic infection. The capacity for gigantism is an important new facet in fungal pathogenesis that provides the pathogen with the ability to escape host defences. We propose that the transition to gigantism can have profound consequences for the host-pathogen interaction including promoting fungal persistence in the host that can translate into latency and disease relapses.

## Introduction

The interaction between a microbe and a host involves a complex response by both the pathogen and the infected individual. The host has multiple defence mechanisms to avoid infection, damage and disease. Microbial pathogens adapt to survive in a host through multiple changes that include signalling pathways that confer the capacity to survive immune-mediated stresses. Both entities, the host and the microbe, interact and each contributes to the outcome of infection [1].

In the case of fungal pathogens, the interaction with the host frequently results in morphological changes. For example, *Candida albicans* forms pseudohyphae and true hyphae during infection, phenomena associated with virulence [2], [3], [4]. Other examples of fungal pathogens that form filaments during infection are *Aspergillus* species and the agents of zygomycosis. In contrast, *Histoplasma capsulatum* and *Blastomyces dermatitidis* manifest a temperature regulated dimorphism, such that at ambient temperatures they form filaments and at 37°C transform into yeast cells [5], [6], [7]. Although the role of these morphological transitions is not completely understood, it is believed that the phenomenon of fungal dimorphism plays an important function during the interaction of each of these microbes with their host.

The fungus *Cryptococcus neoformans* is the causative agent of cryptococcosis, a disease responsible for over 600,000 deaths per year, which makes this pathogen a major global threat. Cryptococcosis is currently the fourth leading cause of death from infectious diseases in Sub-Saharan Africa [8]. *C. neoformans* is unique among the major fungal pathogens in that it possesses a polysaccharide capsule surrounding a yeast cell body [9]. Capsular polysaccharides are also released into host tissues [10], [11], [12], where they mediate numerous deleterious effects on host immune function [13], [14], [15]. In fact, the polysaccharide capsule is the factor that makes the greatest contribution to the virulence of *C. neoformans* [16].

Although *C. neoformans* can form pseudohyphae during mating [9], this pathogen is mainly found in host tissues as round yeast cells. However, there is a specific morphological change associated with cryptococcal infection that involves a significant increase in capsule volume. Capsule size in *C. neoformans* depends on the growth condition (reviewed in [17]). While capsule size is relatively small in standard laboratory media and in the environment, it undergoes a large increase in capsule size during pulmonary infection [18], such that it can comprise more than 90% of the total volume of the cell [19]. Capsular enlargement is believed to confer an advantage to the microorganism during its interaction with the host. For example, capsule growth interferes with complement-mediated phagocytosis [20] and protects the yeast cell against free radicals and antimicrobial agents [21]. Furthermore, increased capsule size makes the yeast more difficult to phagocytose by a variety of phagocytic cells, including amoebas that can prey upon *C. neoformans* in the environment [21].

We now report another morphological change whereby gigantic fungal cells are formed in tissue. This change is achieved, not only by a significant increase in capsule size, but also by an enlargement of the cell body. During pulmonary infection, we observed that a significant proportion of yeasts in the lung had cell volumes 900-fold larger than cells grown in standard laboratory conditions. In retrospect, giant cells have been noted in prior studies [18], [22], [23], [24], [25], but were never isolated or studied. The emergence of fungal giant cells poses a formidable problem for the immune system. In this study we present experimental evidence suggesting that *C. neoformans* gigantism may be a strategy that confers upon the organism the ability to survive within the host for long time periods.

## Results

### *C. neoformans* produce gigantic cells during infection

**Figure 1:**
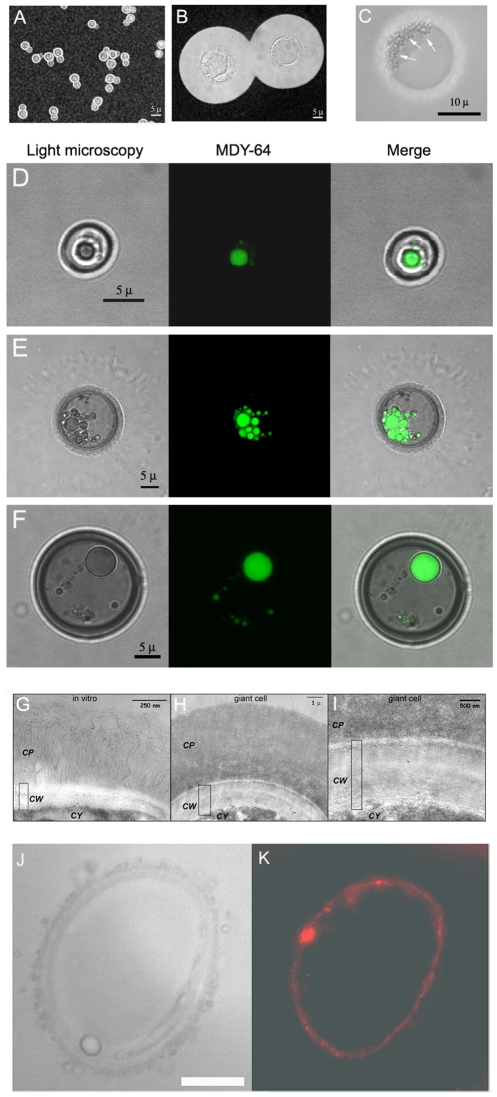
Morphological features of giant cells from infected mice. A) Cells grown in vitro in Sabouraud medium. B) Cell obtained from the lungs of a mouse infected with *C. neoformans* (10^5^ cells/mouse) 5 weeks earlier. C) Photomicrographs of a giant cell illustrating the presence of multiple vesicles (highlighted with arrows) within giant cells. Scale bar, 10 µm. D, E and F, vacuole staining with MDY-64 dye. Yeast cells isolated from the lung of mice four weeks after infection with 10^5^ yeast cells were stained with the specific vacuole marker MDY-64 as described in Materials and Methods. Localization of the signal was performed by confocal microscopy. D) Cell of regular size. E and F) Giant cells. In all the cases, regular light microscopy, fluorescence and the merge of both images are shown. Scale bars in the left panels applies to the corresponding middle and right panels. G, H and I, transmission electron microscopy of the capsule and cell wall of regular and giant cells. Cells grown in vitro or giant cells obtained from the lungs of mice three weeks after infection were fixed and processed for TEM. G, cell grown in vitro; H, giant cell; I, magnification of the cell wall region of the giant cell shown in B. *CP*, capsule; *CW*, cell wall; *CY*, cytoplasm. The rectangle indicates the width of the cell wall. J,K, staining of giant cells with anti-melanin mAb. J, light microscopy; K, fluorescence. Scale bar, 10 µm.

While the typical size of *C. neoformans* cells ranges between 4–8 microns (Figure 1A), we confirm the existence and report the recovery of *C. neoformans* cells of enormous size formed during infection (Figure 1B). Although these cells manifested a large increase in capsule size, there was also a concomitant increase in cell body size. The cell body reached 25–30 µm in diameter, which was almost 7-fold greater than the 4.5 µm average size observed in vitro. This effect was more dramatic if the size of the capsule was included, with the giant cell size typically ranging from 40 to 60 µm in diameter, although extremely large cells with diameters around 70–100 µm were occasionally observed. If one considers volume applying the formula for a sphere (V = 4/3×π×(r^3^)), then giant cell formation involved an increase of 900-fold in cellular volume, compared to cells grown in Sabouraud medium. We investigated whether the phenomenon was found in different cryptococcal strains. Consequently, we infected different individual mice with ten different *C. neoformans* strains (both serotype A and D, including standard strains, such as H99, 24067 or B3501, and clinical isolates from the Yeast Collection of the Spanish Mycology Reference Laboratory). For all strains, we found giant cells after three weeks of infection, indicating that this phenomenon applied to diverse strains (results not shown). Hence, we focused our efforts on the model serotype A strain H99, and we arbitrarily defined giant cells as those with a cell diameter greater than 30 µm (capsule included), a size that is 5–6 times the usual size observed in vitro, and is virtually never encountered during in vitro experimental conditions. Using this strain, we observed giant cell formation in four different mouse strains (CD1, BALB/c, C57BL/6J and CBA/J), indicating that the emergence of giant cells was also not mouse strain specific.

### Morphological features of the giant cells

Giant cells had different cellular features than cells of regular size (Figure 1). Giant cells frequently contained multiple vesicles of unknown function that could reach more than 50 per cell (Figure 1C). In addition, there was usually a single enlarged vesicle that occupied a significant proportion of the cell body volume. To better identify these intracellular structures, we stained the cells with the vacuole specific marker MDY-64. In regular cells, we normally observed the presence of a single vacuole (Figure 1D). In giant cells, we observed two patterns of staining with this specific marker (Figure 1E,F). Multiple vesicles which stained with the vacuole marker were identified in approximately 50% of giant cells, whereas the remainder displayed staining mainly in a single large intracellular vesicle. These results suggest that in some giant cells, the vacuole fragmented into multiple vesicles or the smaller vesicles failed to coalesce.

A peculiarity of the giant cells was the abnormally large width of their cell wall. This feature was most apparent when the cells were observed by transmission electron microscopy. Using this technique, we could determine that the cell wall of regular cells had a width between 50–100 nm (Figure 1G). In contrast, the width in giant cells was 20–30 larger, ranging from 2 to 3 µm (Figure 1H,I). In these pictures, it was also apparent that the density of the capsule differed between regular and giant cells. In the case of yeast obtained in vitro, the cells displayed a low density capsule with individual polysaccharide fibers attached to the cell wall (Figure 1G). In giant cells, the capsule was significantly denser in the regions close to the cell wall (Figure 1H,I).

Fungal cell suspensions recovered from the lungs of infected mice had a dark brownish colour. We hypothesized that this phenomenon could be due to *in vivo* pigment accumulation at the cell wall level, in particular melanin [26]. To investigate this hypothesis, we stained giant cells with specific mAbs to melanin [27]. Giant *C. neoformans* cells bound mAb to melanin at the cell wall level (Figure 1J,K), suggesting that this structure was melanized. In addition, we observed that giant cells showed a high degree of autofluorescence (result not shown), which has been reported in cryptococcal cells grown in certain media [28].

**Figure 2:**
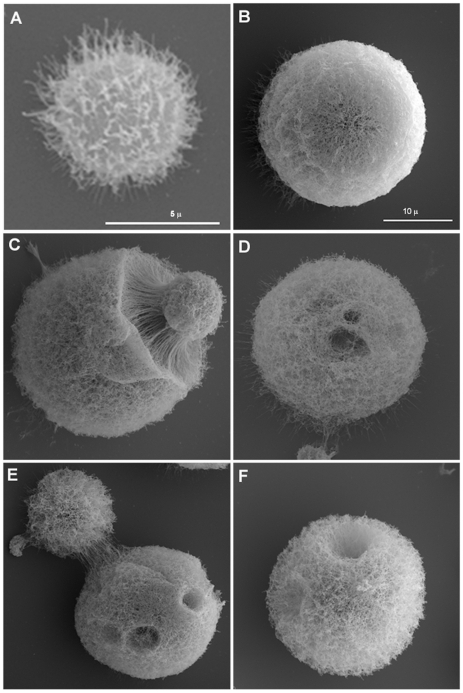
Scanning electron microscopy of cells grown in vitro and of giant cells. A) Cell in vitro grown in Sabouraud. B-F) Giant cells isolated from lung. Scale bar in panel B (10 µm) also applies to panels C-F.

Scanning electron microscopy images suggested that the capsule of giant cells was different from that of cells grown in vitro. For cells grown in standard Sabouraud medium we noted that the dehydration and fixing process resulted in polysaccharide shrinkage and aggregation of fibers such that regions of the cell wall became exposed (Figure 2A). In contrast, the architecture of the capsule of giant cells appeared intact and well preserved, revealing a highly cross-linked polysaccharide net (Figure 2B) that accumulated around the cell body as a very compacted layer (Figure 2C). In addition, we frequently observed the presence of “holes” in the capsule (Figure 2 D, E and F), which we interpreted as pathways formed during recent budding. Transmission electron microscopy images confirmed that the capsule of giant cells was denser than the capsule of in vitro cultivated cells (Figure 1 H, I).

**Figure 3:**
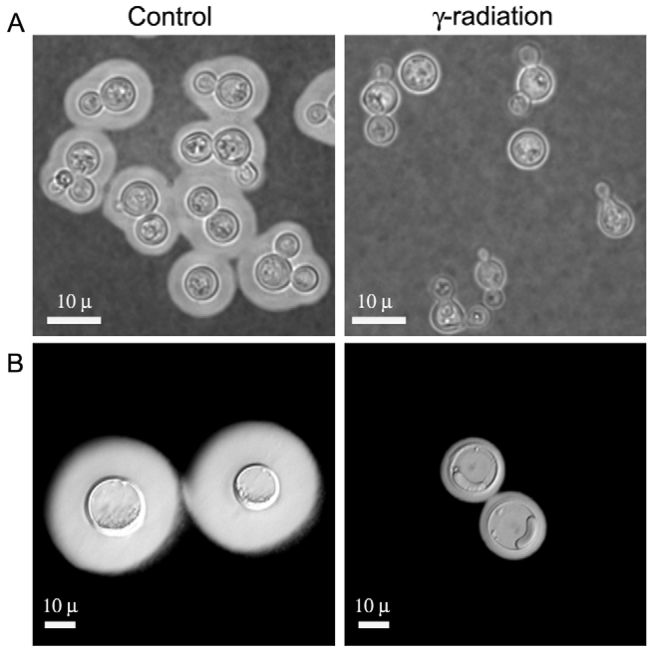
Capsule release by γ-radiation. Cells of in vitro-enlarged capsule (A) and giant cells (B) were exposed to γ-irradiation, suspended in India ink, and observed under the microscope. Images of representative cells are shown before (control, left panel) and after irradiation (γ-irradiation, right panel).

The higher degree of cross-linking in the capsule of giant cells relative to in vitro grown cells was confirmed by treating the cells with DMSO or γ-radiation, procedures known to strip the capsule of cells of regular size [29], [30], [31]. γ-radiation removed the majority of capsule of giant cells, but the inner region of the capsule remained attached to the cell (Figure 3). In contrast, DMSO treatment did not affect capsular size of giant cells while it routinely strips the capsule of cells grown *in vitro* (results not shown). The increased resistance of the inner capsule to radiation and the overall capsule to organic solvents is consistent with a higher degree of capsular cross-linking by the giant cells.

**Figure 4:**
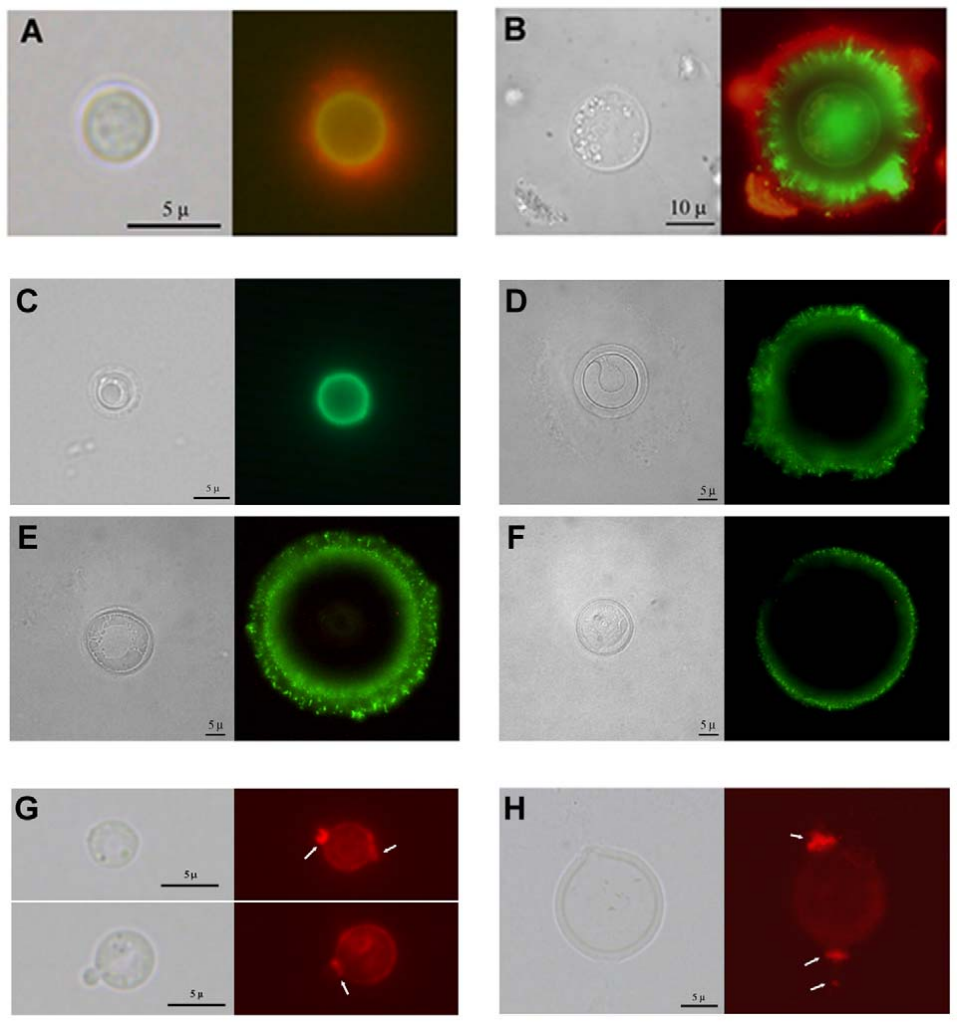
Differences in capsular structure between regular and giant cells. Yeast cells from lung extracts were incubated in mouse serum and labelled with antibodies to C3 and GXM. A) cell of a regular size; B) giant cell. Light microscopy and complement localization (green fluorescence) and capsule edge (red fluorescence) are shown. C–H) capsular features shown by fluorescence. C–F) Indirect immunofluorescence with GXM-binding mAbs. MAb 18B7 labelling of cells of regular size (C) or giant cells (D–F) isolated from lung of infected mice. Light microscopy and fluorescence pictures are shown for each cell. G–H) Binding of wheat germ agglutinin to *C. neoformans*. The presence of chitin-like structures in the capsule was studied by the binding of WGA to the cells as described in Materials and Methods. G) Cells grown in Sabouraud and then transferred to 10% Saboraud buffered at pH 7.3 with 50 mM MOPS buffer to induce capsule enlargement. H) A representative giant cell isolated from the lungs of infected mice. Arrows indicate the major regions where WGA binds to the cells. Scale bars in C–D in light microscopy panels denote 5 microns and apply to the fluorescence images.

In *C. neoformans*, complement deposition is affected by the porosity and blocking capacity of the capsule [32]. Consequently, we characterized complement localization in the capsule of giant cells as a measure of capsule penetrability. As shown in Figure 4A, complement is known to deposit in the inner location of the capsule near the cell wall of typical yeast cells [20]. When giant cells were incubated in mouse serum, we observed that complement was not detected in the inner regions of the capsule (Figure 4B). The exclusion of complement from the inner capsule is consistent with reduced permeability resulting from increased fibril cross-linking.

To ascertain whether giant cells manifested antigenic differences from cells grown in vitro we used indirect immunofluorescence with mAb 18B7. We compared cells of different size obtained from the lungs of infected mice, to avoid the possibility that factors of the immune system influenced the antigenic properties of the capsule. When stained with mAbs 18B7, cells of small size exhibited a uniform annular binding pattern (Figure 4C), which was identical to the binding of this mAb to cells grown in vitro [32]. In contrast, most of the fluorescence localized to the edge of the giant cell capsule, and this binding was diffuse and punctate (Figure 4D–F). Moreover, many cells showed a double ring, punctate pattern, with a more uniform inner ring and a rougher, more diffuse outer ring.

Chitin-like structures in the capsule were recently demonstrated by the specific binding of fluorescent wheat germ agglutinin, which binds to sialic acids and β-1,4-N-acetylglucosamine (GlcNAc) oligomers [33]. We used WGA to ascertain whether these structures were also present in giant cells. Cells grown in vitro bound WGA, especially at the neck between the mother cell and the bud (Figure 4G). In giant cells, these structures were particularly prominent. Protrusions into the capsule were longer, reaching several microns (Figure 4H).

### Replication of giant cells

**Figure 5:**
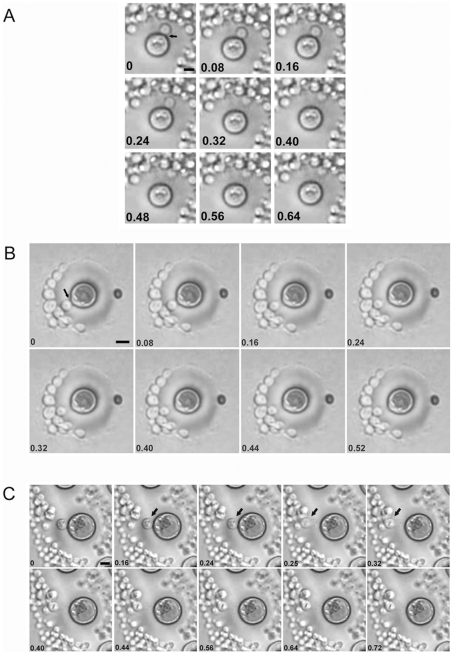
Time lapse images of replicating giant cells in vitro. Giant cells were obtained from lungs of four weeks-infected mice and placed on Sabouraud plates. After 18 h, the growing colonies were monitored under the microscope. The digital videos were processed with Windows Movie Maker software. This software allowed the conversion of the videos into single pictures, each one represented as a different frame of the video. The number in each picture corresponds to the time in seconds. A, B and C correspond to three different budding events. Corresponding videos are included as supporting information files.

Giant cells were viable, since they replicated when placed on fresh agar plates. Daughter cells emerging from giant cells were not trapped inside the thick polysaccharide capsule, but rather traversed it in less than 0.08 seconds (Figure 5A and B, supporting Videos S1 and S2). In some cells, movement through the capsule was much faster, taking less than 0.01 seconds (Figure 5C, supporting Video S3). By measuring the distance travelled through the capsule by emerging buds and the transit time we estimated that the daughter cells traversed the capsule at 20–50 m/h, which is a remarkably high velocity for a microscopic unicellular particle in a gelatinous environment. This data suggested the existence of a motive force propelling and separating the buds from the mother cell's capsule. We observed that giant cells could produce several daughter cells over brief periods of time (2–3 hours), with buds always emerging from the same cell site (data not shown). Despite ejection, the daughter cells remained close to the capsule of the mother cells where they replicated, producing abundant progeny around the giant cells. Even after the giant cells were surrounded by daughter cells, new buds were still ejected with significant force, since they were able to displace and move the surrounding cells upon impact (see supporting Video S4).

When placed on agar, we observed that not all giant cells produced daughter cells after 24 h, suggesting that some of these cells were metabolically arrested, or had died prior to or during the isolation procedure. To measure the percentage of replicating cells we obtained giant cells from two mice and counted the proportion of giant cells producing colonies after 24 h on agar with a microscope. The percentage of giant cells reproducing was 60% and 73% for each mouse, respectively, indicating that the majority of giant cells were viable.

As a secondary technique for testing giant cell viability we used the method based on the reduction of 2,3-bis(2-methoxy-4-nitro-5-sulfophenyl)-2H-tetrazolium-5-carboxanilide inner salt (XTT) by alive cells. Giant cells manifested a strong capacity to reduce XTT, which was approximately 100-fold greater than the activity shown by the same number of cells grown in vitro (data not shown). This result indicates that giant cells are metabolically active.

### In vitro cellular growth in minimal media and its relation to cell aging

**Figure 6:**
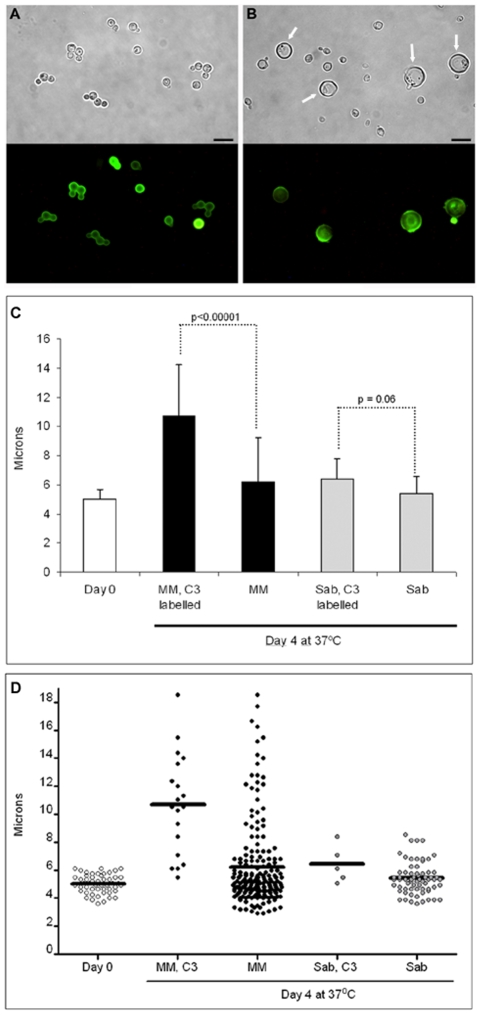
Giant cell formation in vitro. Cells from H99 strain were grown in Sabouraud, washed and labelled with mouse serum. C3 deposition was then detected by immunofluorescence (A). After incubation in mouse serum, cryptococcal cells were transferred to minimal medium (MM) for four days, and C3 was detected (B). Scale bar in A and B, 10 µm, and apply to the corresponding fluorescence panels. C) Cells were grown and labelled as in A (Day 0 sample), transferred to Sabouraud or MM for 4 days at 37°C, and the cell size was measured in the C3 labelled population (“MM, C3 labelled” and “Sab, C3 labelled”) and in the whole population, including both C3 labelled and unlabelled cells. The average and the standard deviation (error bars) are plotted and p-values for the highlighted comparisons are shown. At least, 20–50 cells were counted, except in the “Sab, C3 labelled” samples, where C3 positive cells were rarely identified due to the overgrowth of the culture. Kruskall-Wallis test was used to assess statistical differences. D) Scatter representation showing all the cells plotted in panel B. The line in each sample denotes the average of the distribution.

We tried to induce giant cell formation in vitro by incubating the cells in different media. When we incubated the cells in minimal media, around 4–5% of the cells showed a marked increased in cell size over 4 days. These cells reached up to 25–30 µm in diameter (capsule included), approximating, but not quite reaching the size of the giant cells recovered from mouse lungs. In addition, these in vitro giant cells showed other phenotypic differences with the giant cells obtained in vitro, such a smaller capsular size and a lack of enlargement of the cell wall (result not shown). Although this in vitro medium only partially reproduced the gigantism phenomenon, we used it to study if there was any relationship between cellular enlargement and the age of the cells. We hypothesized that massive cellular growth required a prolonged period of time, so the majority of the giant cells obtained would be originated from the initial inocula. To explore this hypothesis, we labelled the cells with complement. Complement proteins, especially C3, bind to the capsule covalently without inhibiting cell growth and do not segregate to buds after replication [34]. Consequently, we labelled cells grown in Sabouraud medium with mouse C3 and then incubated them in minimal medium (which induces a small population of giant-like cells) and Sabouraud medium. At time zero, all the cells were labelled with complement (Figure 6A), but after 4 days of incubation in minimal medium, only a few cells remained labelled (Figure 6B). When we measured the average size of the cells with complement bound after four days of incubation in minimal medium, we found that these cells had a significant larger size than the cells incubated in Sabouraud medium (Figure 6B). We repeated this experiment, placing the cells in parallel in minimal medium, which induces cell enlargement in some cells, and in Sabouraud medium, in which cell enlargement is not expected. Then, we measured cell size of complement-labelled and non-labelled cells. In minimal medium, we observed that the complement-stained cells were significantly larger than the unlabelled population (Figure 6C, D). Large cells were not found in Sabouraud medium after C3 labelling and there was no difference in the size of cells with and without C3 labelling (Figure 6C, D). This result indicates that cellular enlargement and giant cell formation is correlated with the age of the cells, such that the giant cells are the older cells in the culture.

### DNA content

**Figure 7:**
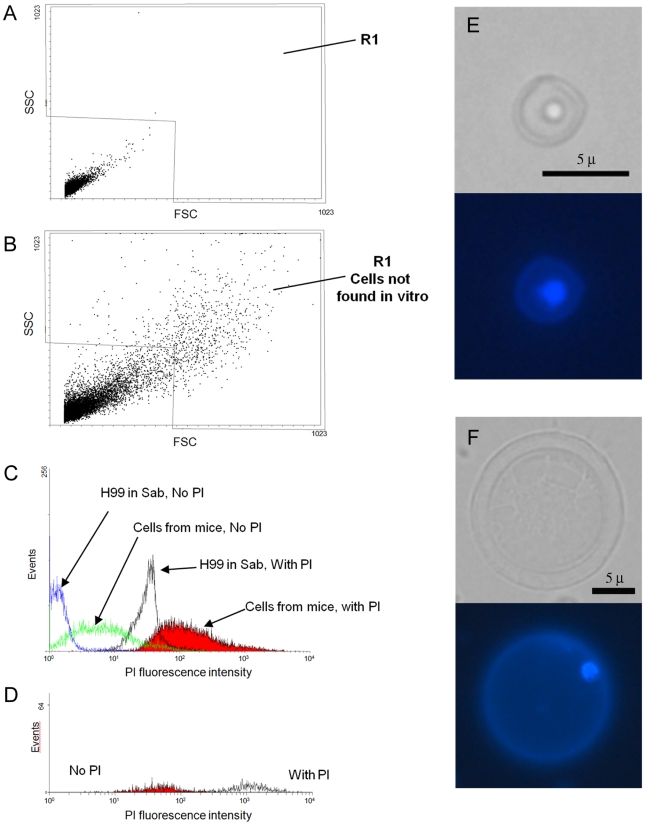
Determination of DNA content by cytometry and DAPI staining. Fungal cell samples were obtained from the lungs of one mouse 3 weeks after challenge with 10^5^ *C. neoformans* cells. As a control, cells grown for 24 hours in Sabouraud were also used. The cells were fixed after incubation at 60°C for 45 minutes. Propidium iodide was immediately added at 10 µg/mL DNA content and the labelling was determined by the propidium iodide fluorescence intensity. Matched samples were subjected to cytometry without propidium iodide. A) FSC/SSC plot of cells grown in Sabouraud. B) FSC/SSC of cryptococcal cells from lungs. Region 1 delimits all the cells whose size and complexity are not found in cultures grown in vitro and presumably cover all the cells with increased cell size. C) Propidium iodide fluorescence intensity of the four samples analyzed: in vivo isolated cells (plus and minus propidium iodide) and in vitro cells (plus and minus propidium iodide). D) Propidium iodide fluorescence intensity of the cells from the lung present in R1. E–F) DAPI staining. Yeast cells obtained from the lungs of mice infected with 10^5^ yeast cells were stained with DAPI. E) cell of regular size; F) giant cell. Corresponding light microscopy and fluorescence images are shown. Scale bar in the light microscopy panels apply to the fluorescence images.

We hypothesized that giant cell formation was a consequence of continued cell cycle progression without cellular fission. In other fungi, cell size can be related to DNA content [35], [36]. Using flow cytometry to measure DNA content, we found that giant cells had low permeability to propidium iodide by regular staining protocols (results not shown), but could be permeabilized by heating at 60°C for 45 minutes. As mentioned above, cells from the lungs of mice infected for 3–4 weeks showed strong autofluorescence, so we measured the intensity of the signal in the presence or absence of propidium iodide. We first analysed the difference in the forward scatter (FSC, cell size) and side scatter (SSC, cell complexity). When we compared these parameters, we observed a population of larger yeasts in the cells isolated from the lung that was not present in the yeast cells obtained in vitro, as was expected with the presence of giant cells in vivo (Figure 7A, B). This population was defined as region 1 (R1), which contained the giant cells present in the population. To estimate the DNA content, we added propidium iodide to these samples. When we measured the propidium iodide staining, we found that there was a high variation in the DNA content in the cell population obtained from lungs (Figure 7C), which implied a high variation in the DNA content of cells in vivo. Fungal cells isolated from the lungs of infected mice also displayed significant autofluorescence in the absence of propidium iodide staining. To assess the staining of giant cells, we subtracted the autofluorescence of the cells present in region 1 from the fluorescence value determined in the presence of propidium iodide. The mean fluorescence intensity for the giant cells was almost 10^3^ fold higher than the signal measured in cells grown in vitro (Figure 7D). This result suggested that giant cells contain multiple copies of DNA. To quantify the ploidy level of the giant cells further, we performed real time PCR to amplify the ITS1 region from ribosomal DNA. We included controls of purified genomic DNA of known concentrations, which yielded a lineal relationship between crossing point values and DNA concentration. When we compared 300 giant and regular cells there was a difference in the Ct values of more than 3 cycles (36.95 in giant cells versus 40.00 in regular in vitro grown cells). When we estimated the amount of DNA present in each condition according to a standard curve generated using different concentrations of genomic DNA, we calculated that the amount of DNA in each giant cell was 1.3×10^−7^ ng. In contrast, in vitro cultivated cells contained 8.4×10^−9^ ng. This result indicated that giant cells contained 16× DNA than regular cells. This experiment was repeated using different cell concentrations and consistent results were obtained (result not shown).

DAPI was used to directly observe the nucleus of the giant cells. This staining revealed that both regular (Figure 7E) and giant cells (Figure 7F) contained a single nucleus.

### Signal transduction pathways involved in giant cell formation

**Figure 8:**
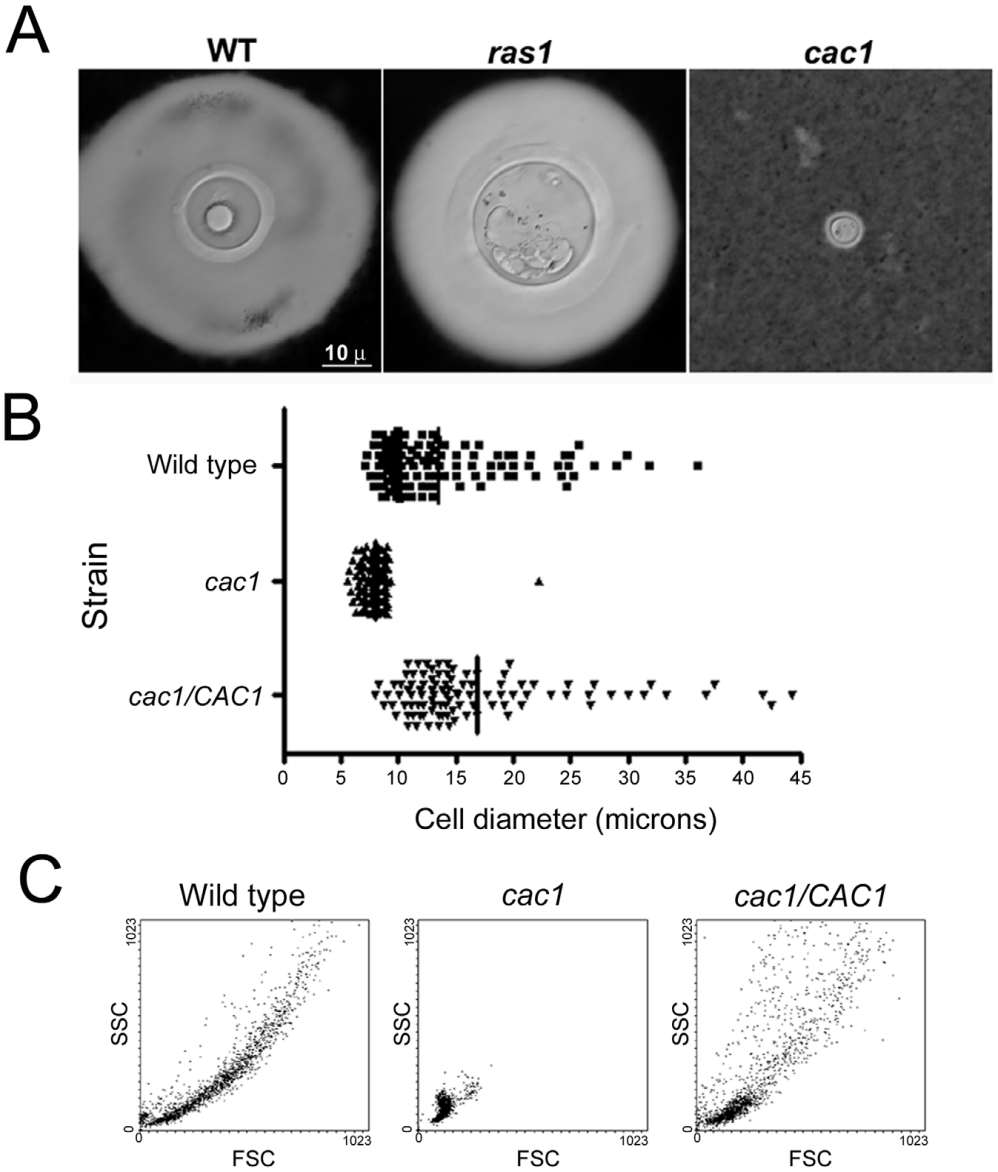
Giant cell formation depends on cAMP, but not Ras1. C57BL/6J mice were infected with H99, *ras1* (Ras1 mutant) and *cac1* (adenylate cyclase mutant) strains (10^6^ per mouse). After three weeks, the mice were sacrificed and fungal cells were isolated. Representative pictures of fungal cells are shown. A) India ink microscopy, scale bar in left panel applies to all the pictures. B) Cell size distribution of wild type, *cac1* mutant and its reconstituted strain (*cac1/CAC1*) after 5 days of growth in minimal medium at 37°C. The cells were suspended in India Ink to delimit the capsule, and the diameter of the cells (capsule included) was measured microscopically. C) Forward Scatter/Side Scatter plot of *cac1* mutants after incubation in minimal medium. The cells described in B were analysed by flow cytometry to obtain the corresponding forward scatter (FSC, correlated with cell size) and side scatter (SSC, correlated with cell complexity).

We investigated the potential involvement of two of the major signal transduction pathways in *C. neoformans* (cAMP and Ras1 [37], [38]) in giant cell formation. Ras1-deficient cells produced giant cells in the lungs of infected mice (Figure 8A). In contrast, mutants unable to accumulate cAMP (lacking adenylate cyclase encoded by the *CAC1* gene) did not produce giant cells during murine infection (Figure 8A), suggesting that this pathway was required for giant cell formation.

The absence of giant cells in mice infected with the cAMP mutant was associated with a reduced fungal burden. Hence, the lack of giant cells with this mutant in vivo might be related to the ability of the host to rapidly clear the fungus. For this reason, we examined whether a *cac1* mutant formed giant cells in minimal media. The *cac1* mutant failed to produce giant cells, whereas a significant proportion of cells of the wild type (H99) and reconstituted (*cac1/CAC1*) strains manifested cellular enlargement (Figure 8B), confirming that cAMP pathway is involved in giant cell formation. To further characterize this phenotype, we analysed the forward and side scatter profile of the cells from the three strains by flow cytometry, since this type of plot allows for clear differentiations in cell size. As shown in Figure 8C, the *cac1* mutant yielded a very homogenous population of relatively small FSC and SSC values. In contrast, the wild type and reconstituted strains produced more heterogeneous populations, in which cells with larger FSC and SSC values were measured, indicating the appearance of cells of larger size.

### Distribution of fungal cell size during infection and correlation with inflammation

The distribution of cell sizes in vivo was extremely variable depending on the experimental conditions. Under our standard conditions (infection of 6–8 weeks old mice with 10^5^ yeast cells) we consistently found that the proportion of giant cells in the lung was between 1–10%, with variation between individual mice and between experiments. However, in several experiments, we occasionally found that the proportion of giant cells was higher than 90% of the lung fungal cell population. Curiously, in those experiments where the proportion of giant cells was very high, there were no obvious signs of disease and the mice looked healthy. We decided to investigate this observation in more detail by studying the relationship between inoculum and giant cell formation. We hypothesized that infections with low inocula could reproduce chronic or latent asymptomatic infections. For this purpose, we performed infections with high (10^6^/mouse) or a low dose (10^4^ cells/mouse).

**Figure 9:**
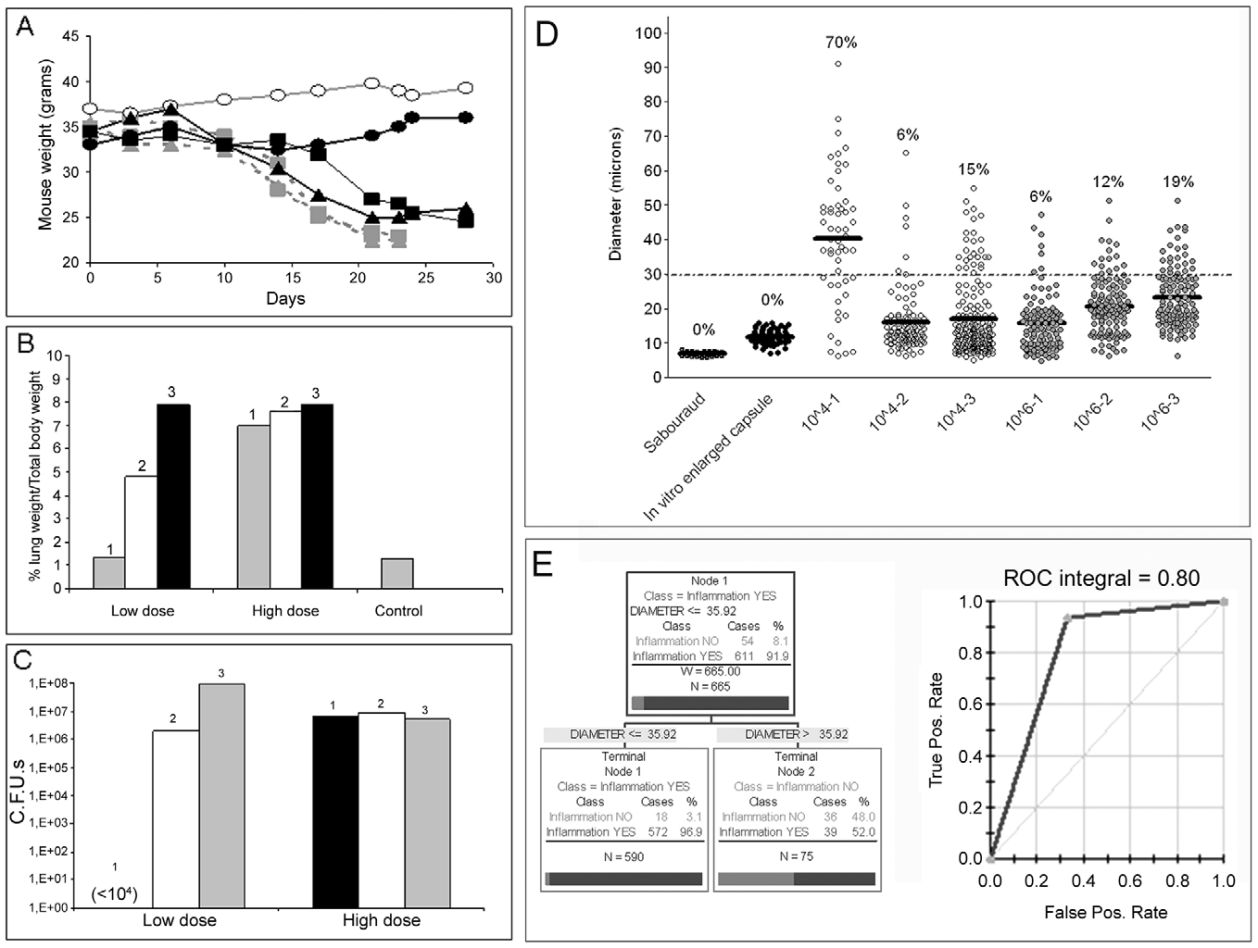
Parameters of disease in mice infected with *C. neoformans* and giant cell proportion in the lungs. CD1 mice were infected with different inocula of *C. neoformans*, and different parameters were monitored during the course of the infection. A) Body weight of mice infected with a low dose (∼10^4^ cells, black line, closed symbols, n = 3) or a high dose (10^6^, grey line, grey symbols, n = 3) of *C. neoformans*. A control mouse, injected with PBS, is also shown (grey line, open symbols). B) Proportion of the lung weight in respect to body weight, as an indicator of lung inflammation. C) CFUs from the lungs of mice described in A and B. D) Cell size of *C. neoformans* in vitro and in vivo. Cell size (capsule included) was measured in fungal samples obtained from mice infected with high or low *C. neoformans* doses (see Figure 9A-C). As a control, cell size was measured in cells grown in vitro in Sabouraud or in 10% Sabouraud buffered at pH 7 with 50 mM MOPS to induce capsule enlargement. The number expresses the proportion of giant cells in each sample, defined as cell with a diameter above 30 µm. The line in each distribution represents the average of the population. E) Results of the CART analysis. Left panel, prediction of the correlation between inflammation and total fungal cell size. A cell size lower than 35.92 µm was strongly associated with increased inflammation. Right panel, corresponding ROC curve, showing a region under the curve of 0.8.

Mice infected with high inocula consistently developed typical cryptococcal disease, as indirectly shown by progressive weight loss (Figure 9A). However, we found a high variation in outcome when the mice were infected with a low inoculum. Most of these mice (2 of 3) developed disease comparable with that seen after infection with a high dose. Severe disease was characterized by dense inflammation in the lungs, increasing the size and weight of these organs (1.3–1.8 grams) such that they accounted for 7–8% of the total body weight (Figure 9B). In contrast, the lung mass of asymptomatic mice (control mice and one of the mice infected with low inocula) was approximately 0.45 grams and ∼1% of total body weight. As expected, mice developing severe disease had a significantly higher number of CFUs (>10^6^/lung) than the mice that did not manifest obvious signs of disease, where the number of CFUs remained very low (<10^4^) during the experiment (Figure 9C). When we recovered the fungal cells from the lungs of these mice, we found profound differences in the size of the yeast cells. The average cryptococcal cell size in mice receiving a high inoculum was around 15–20 µm, which was significantly larger than the size reached when grown in vitro in rich medium (Figure 9D, Sabouraud medium). Although approximately 5–20% of the yeast cells met criteria for giant cells, the enlargement of the majority of cells isolated was mainly due to increase in the capsule size, so the size of these populations was only slightly different from the size reached when capsule size is induced overnight in vitro (see Figure 9D, in vitro enlarged capsule size). We were able to isolate around 1-3×10^3^ *C. neoformans* cells from the lungs of asymptomatic, low dose infected mice and the average yeast cell size was around 40 µm (Figure 9D). Notably, approximately 70% of the isolated yeast cells were giant forms.

We also analysed the proportion of giant cells in the lungs using Classification and Regression Trees (CART) analysis. Using this approach, we found that there was a strong association between the total fungal cell size in the lungs and the degree of inflammation, such that high inflammation was predicted when the average fungal cell size was below 36 µm (Figure 9E). This prediction is in accordance with our initial criteria of defining giant cells as those with a cell diameter greater than 30 µm. When we plotted the corresponding ROC curve, we found that the region under the curve was 0.80, which provides strong support for the prediction. On the other hand, the model could not efficiently predict the proportion of giant cells according to inflammation, due to the low number of yeast cells found in lungs without significant inflammation.

**Figure 10:**
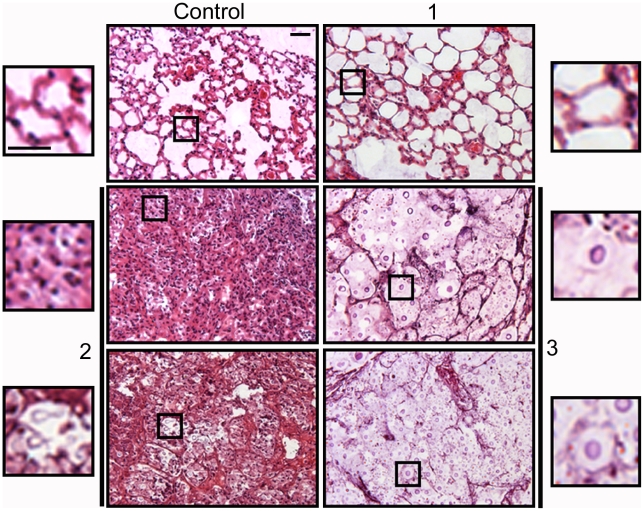
Histological sections of mice infected with different *C. neoformans inocula*. Tissue sections from mice described in Figure 9 were stained with hematoxylin and eosin. Tissue section of control mouse (upper left panel) and three different mice infected with a low *C. neoformans* inocula. Mouse 1, upper right panel; mouse 2, middle and lower left panels; mouse 3, middle and lower right panels (for mice 2 and 3, two different regions are shown). For each panel, a magnification of a region is shown, which is delimited by an inset. Scale bar in upper left magnification, 25 µm, and applies to the rest of amplified panels.

The relationship between the proportion of giant cells and inflammation was confirmed histologically. In control mice, the lungs revealed the typical structure in which alveolar spaces were present throughout the lungs (Figure 10). In the infected mice, we only observed this benign histology in the asymptomatic mouse (mouse 1 of the group infected with 10^4^ yeast cells, Figure 10). In the rest of the low dose (Figure 10) and all of the high dose infected mice (result not shown), dense inflammation was observed and alveolar spaces contained numerous yeast cells and inflammatory cells.

We performed another experiment using older mice (14–16 weeks old), which are more resistant to infection, and a lower infective dose than in the experiment previously described. We infected with either a low (10^3^ cells/mouse, 10 mice) or a high dose (10^5^ cells/mouse, 3 mice), and measured fungal cell size after a month of infection. In this new model, the mice did not develop any visible sign of disease. When the mice were sacrificed after one month, only one of the mice infected with the high dose showed inflammation in the lungs (table 1, 10^5^, mouse 2). In the group infected with low dose, we did not find yeast cells in 3 mice, indicating that the infection had been cleared. In the other seven mice, we found a low number of yeast cells, suggesting a chronic asymptomatic infection. In six of the seven mice, the average size of the fungal cells was above 30 µm, and the proportion of giant cells was between 50–90%. When the mice were infected with a higher dose, the two mice in which no inflammation was detected showed fungal cell sizes above 30 µm, with a proportion of giant cells around 70–80%. In the mouse with inflammation (mouse number 2), the average fungal cell size was smaller (14 µm), and the proportion of giant cells was less than 5%. This data supports the notion that the highest proportion of giant cells is found in hosts with chronic and longstanding infection.

**Table 1:** Fungal cell size and proportion of giant cells in 16-weeks old mice infected with low and high doses of H99 strain.

	Yeast dose (cells/mouse)									
	10^3^							10^5^		
Mouse number	1	2	3	4	5	6	7	1	2	3
Lung weight (mg)	240	390	326	340	339	240	309	550	860	413
Mean ± St. Dev (µm)	34±6	33±6	31±13	20±5	49±9	46±10	44±11	34±12	14±5	40±10
Giant cells (%)	61	74	50	9	91	80	90	67	4	82
Lower 95% CI	32.3	29.5	21.4	15.5	42.1	30.4	39.4	30.1	13.3	34.1
Upper 95% CI	36.3	37.3	40.5	25	57.6	62.4	49.8	37	16	46.1

Ten CD1 mice (16 weeks old) were infected with 10^3^ yeast cells/mouse, and three mice were infected with 10^5^ yeast cells/mouse. The animals were killed after a month, and the weight of the lungs was measured. Fungal cells were isolated as described in Materials and Methods, and the mean value, standard deviation, proportion of giant cells (above 30 µm), and the lower and upper 95% confidence interval were calculated. In three mice of the group infected with a low dose, we did not find any yeast cells, so the data of the other seven mice is represented.

### Susceptibility to oxidative stress

We measured the susceptibility of giant cells to oxidative stress produced by incubation in H_2_O_2_. Giant cells were significantly more resistant to killing by oxidative stress than cryptococcal cells grown in vitro, with survival rates of 19%±10 and 46%±14 for cells grown in vitro and giant cells, respectively (*p* = 0.014).

### In vitro interaction of giant cells with phagocytic cells

To further characterize the interaction of giant cells with host effector cells, we incubated macrophage-like cells with giant cells and observed the outcome of the interaction using live-imaging microscopy. When small-sized cryptococci were exposed to macrophages, we observed rapid and avid phagocytosis, yeast cell transfer between macrophages, fusion of infected macrophages after division, and intracellular replication of the *C. neoformans* cells, as described previously [39], [40], [41], [42], [43] and shown in supporting Videos S5, S6 and S7. None of these phenomena were observed when macrophage-like cells were exposed to *C. neoformans* giant cells, indicating that the interaction between these fungal cells and macrophages was different and had different outcomes. Although in some cases the macrophages seemed to adhere to the giant fungal cells, there was no phagocytosis or macrophage fusion after division (supporting Videos S8 and S9), indicating that macrophages could not cope with the giant cells.

## Discussion

*Cryptococcus neoformans* giant cells have been occasionally described in the literature primarily as curiosities in histological tissue sections [22], [23], [24], but their importance in pathogenesis has remained obscure. Apart from the fundamental problems in cell biology posed by the mechanisms responsible for the transition to gigantism, we considered that the presence of fungal giant cells would pose a major problem for the immune system simply by virtue of their size.

Giant cell formation was associated with several changes to the capsule relative to the typical cells observed in vitro. These changes represented an exaggerated response in capsule, cell body, and cell wall size during infection. Moreover, the resistance to capsule shedding after γ-radiation exposure suggests a more compact and dense structure. Such an increase in capsular compactness could confer a survival advantage in vivo since the capsule is known to protect against oxidative fluxes of the types produced by immune effector cells [21]. Consistent with this idea, we have observed that giant cells are more resistant to oxidative stress.

Giant cells maintained their enormous size ex vivo, although they produced smaller cells in agar at replication rates similar to those observed in vitro. Another remarkable aspect of the budding process is the rapidity with which the buds traversed the capsule, especially considering the denseness and compactness of the polysaccharide noted by scanning and transmission electron microscopy. However, we did observe holes in the capsules of giant cells with dimensions that approximated the size needed for daughter cells to emerge. Similar capsule holes have been occasionally described in encapsulated cells grown in vitro [44]. The strong binding of WGA to these cells also suggests the presence of chitin-like structures, which have been proposed to be involved in the movement of the bud through the capsule of the mother cell [33]. Given prior work noting tunnel-like structures formed around buds [33], [34], it is possible that the rapid egress of buds from the mother cells represent movement along such structures that provide a non-obstructed conduit through the capsule.

The cellular mechanisms by which cryptococcal cells enlarge to gigantic sizes are not known and a complete understanding of this phenomenon is beyond the scope of the current work. Nevertheless, we explored the potential mechanism of cell division without fission as a way for progressively increasing mass. Cell growth is intimately dependant on the cell cycle. The cells need to reach a critical size for cell cycle to progress, and there is a constant ratio between the mass of the cell and its DNA content (reviewed in [45], [46], [47], [48]). In plants, the phenomenon is striking, because their cells can enlarge in size by 100- or even 1000-fold, and this is achieved by endoreduplication, which is the process in which the cell increases the ploidy of the cells through several rounds of DNA replication [49]. Recently, it has been shown that bacteria from the genus *Epulopiscium*, which grow to lengths of 200–300 µm and widths of 40–50 µm, have extreme polyploidy, generating tens of thousands of copies of their genome [50]. In a process that may be relevant to cryptococcal gigantism, there are some symbiotic bacteria that undergo an important differentiation process achieved by genome amplification by endoreduplication in plant nodules resulting in significant cell enlargement [51] and provide beautiful examples of how some factors of symbiotic plants regulate the cell cycle of their symbiotic microorganisms.

We hypothesized that giant *C. neoformans* cells achieved their size by repeatedly entering G1 cycles without dividing. To investigate this possibility we stained cells for DNA. Cells recovered from infected animals produced a noisy FACS profile that was interpreted as being consistent with cell-to-cell variation in DNA content. By analyzing cell size and fluorescence intensity we showed that cell size correlated with DNA content, thus establishing that larger cells have more DNA, a result confirmed by real-time PCR. These findings are consistent with a mechanism for DNA replication without cell fission.

The proportion of *C. neoformans* giant cells in infected mouse lung was a function of total microbial burden and pulmonary inflammation. Okagaki et al also found differences in giant/titan cell proportions during infection experiments with MATa and MATα strains, with the proportion of titan cells being higher when mice were co-infected with both mating types (Okagaki et al, see related article in the current PLoS Pathogens issue). These authors have concluded that titan cell formation is induced by the pheromone signalling pathway. Taking our and their results together, we can conclude that gigantism is a morphological response to host environments that impact cAMP and pheromone signalling pathways, which could regulate the cell cycle with the final purpose of generating giant cells during infection. The fact that the survival of the host is not compromised when the proportion of giant cells is high suggests that giant cells can survive in a local environment in the host for protracted periods of time without disseminating in the setting of intact host immunity, a finding in agreement with Okagaki's report. This notion is consistent with reports that a moderate increase in cell size due to capsule enlargement interferes with *C. neoformans* dissemination from the lung [52], [53].

Various studies have suggested that *C. neoformans* dissemination is associated with intracellular survival inside macrophages [54], [55], [56], [57], but this model cannot be applied to giant cells since they exceed the size of macrophages. Hence, the increased size of the giant cells is likely to be an impediment for their dissemination as they are simply too large to cross biological barriers and/or transverse capillary diameters. Nevertheless, such cells are viable and capable of producing small sized variants when placed in suitable conditions, such as rich agar. Taken together, our findings suggest that giant cell formation could provide the fungus with a strategy for prolonged survival in a host. Cells could conceivably survive through the life of the host and then return to soils when an animal dies. Alternatively, the giant cells could await permissive host conditions such as in the setting of advanced HIV infection, immunosuppression after organ transplant, or other conditions impairing host immune responses that lead the fungus to proliferate to produce abundant progeny. In this context, it is noteworthy that micro-yeast forms have been described in the lung of infected mice [18], [58], suggesting another form of size polymorphism at the other end of the scale. All these findings indicate that during infection, *C. neoformans* can display a wide variation in cell sizes, ranging from micro-forms to giant cells, and suggest that each of these morphotypes have different roles in the pathogenesis of persistence and dissemination.

We are aware of occasional reports of gigantic cells in other fungal species. For example, giant *Candida albicans* cells with diameter up to 30 µm have been described [59], [60], and similar large cells have been described for other pathogenic fungi during infection. The arthroconidia of *Coccidioides immitis* and *Coccidioides posadasii* swell to form giant spherules (typically 30–150 µm in diameter) during mammalian infection and the spherules produce a large number of endospores derived from the cell membrane, each with a single nucleus [35]. When the spherule is mature, the cell membrane is dissolved and the endospores are released. Another example of fungal giant cells occurs in species from the genus *Emmonsia*, responsible for adiaspiromycosis in humans. *Emmonsia crescent* cells reach up to 200–700 µm during infection and these forms are multinucleate. *Emmonsia parva* forms cells up to 40 µm, and they also contain several nuclei [36]. It is possible that gigantism is a general property of unicellular fungi that is expressed under certain conditions. If this is the case, the reproducibility of giant cell formation during cryptococcal infection provides an excellent experimental system for the study of this phenomenon.

In summary, *C. neoformans* cells can achieve gigantic dimensions during infection and the phenomenon suggests that gigantism may be considered a new form of fungal dimorphism. The occurrence of extraordinarily large cells may enable an adaptation for persistence in certain hosts. The findings for *C. neoformans* together with the similar reports in other fungi suggest that this may be a general mechanism for fungal survival under certain environments and possibly contribute to persistence during host-pathogen interactions.

## Materials and Methods

### Yeast strains and growth media

For most experiments, serotype A H99 strain was used [61]. In some experiments the following strains were also used: 24067 (serotype D, ATCC); B3501 (serotype D, [62]); RPC3 (*cac1::URA5*, [37]); RPC7 (*cac1::URA5/CAC1*, [37]); LCC1 (*ras1::ADE2*, [38]) and different clinical isolates from the Yeast Collection of the Spanish Mycology Reference Laboratory (CL2132, CL4860, CL5154, CL5632, CL5707, CL5066 and CL4979). Yeasts were grown in Sabouraud liquid medium at 30°C with moderate shaking (150 r.p.m.). In some cases, the yeast cells were grown in minimal media (29.4 mM KH_2_PO_4_, 10 mM MgSO_4_, 13 mM Glycine, 3 µM thiamine, 15 mM glucose, pH 5.5). For melanization, L-DOPA containing medium was prepared as in [63]. In other experiments, the cells were transfer from the original Sabouraud culture to 10% Sabouraud medium pH 7.3 with 50 mM MOPS buffer, as described in [17], to induce capsule enlargement in vitro.

### Mouse strains and infection models

Six to eight weeks old female BALB/c, C57BL/6J (Jackson Laboratories, Bethesda, MD) and CD1 mice (Charles River Laboratories) were used in this study. In some experiments, older CD1 mice (16 weeks old) were also used. *C. neoformans* strains were grown at 30°C, washed with sterile PBS, and suspended at specific cell densities. Fifty microliters of the selected yeast cell suspension were injected intratracheally into mice previously anesthetized with a xylazine/ketamine mixture, as described [64].

### Histology sections of lung tissues

Lungs were excised from mice at different infection times and fixed in formalin for 48 h at room temperature. The tissues were then dehydrated and embedded in paraffin using an STP120 Tissue Processor (Microm International, Walldorf, Germany). Then, 5 µm tissue sections were obtained using a Leica RM2245 microtome and placed on glass slides. Hematoxylin/eosin staining of the tissue sections was performed using standard protocols.

### Fungal cells isolation from lungs of infected mice

Mice were euthanized at different times after infection and the lungs were removed. Lung tissue was then homogenized in 10 mL of PBS with 1 mg/ml collagenase (Roche, Mannheim, Germany). The cell suspension was incubated for 1 h at 37°C with occasional vortex agitation, and washed several times with sterile distilled water. The cells were suspended in sterile distilled water, and immediately placed in fixative for microscopy, in fresh medium for microscopy observation, or in Sabouraud agar at 30°C overnight to observe in vitro budding.

### Microscopy techniques

Cells were viewed with different microscopes. In some experiments, an Olympus AX70 microscope was used and pictures were taken with a digital camera using QCapture Suite V2.46 software for Windows. Alternatively, a Leica DMI3000B connected to a DFC300 digital camera with LAS 3.3.1 software, or a Leica DMI 4000B or a Leica DMRD microscope connected to a Leica DC200 digital camera with IM1000 software were used. To visualize the size of the capsule, the cells were mixed with an India ink suspension. Digital Images were processed with Adobe Photoshop 7.0 software (San Jose, CA). For confocal microscopy, a SP5 confocal microscope (Leica Microsystems) was use.

### Macrophage-like cell lines and cell culture techniques

The macrophage-like cell line RAW264.7 was maintained in DMEM medium supplemented with 10% heat-inactivated fetal bovine serum, 10% NTCT, and 1% of non-essential amino acids at 37°C in the presence of a 5% CO_2_ atmosphere.

### Live-imaging of the interaction between macrophages and fungal cells

For phagocytosis experiments, 5×10^4^ macrophages were placed on 96-well plates and incubated overnight at 37°C in the presence of 5% CO_2_, so that a total number of 10^5^ macrophages was expected after this incubation given a phagocytic cell replication time of approximately 12 h. Fungal cells were added at a 1∶2 (macrophage:yeast cells) ratio in 200 µL of medium. Yeast cells of regular size were obtained by growing in Sabouraud medium overnight. To isolate giant cells, fungal cells were isolated from the lungs of infected mice as described above. Giant cells were separated from the rest of the fungal population by passing the sample through 11 µm filters. Although we defined giant cells as those larger than 30 µm (capsule included), we observed that the capsule did not contribute to retention on the filters, since the size of the cell delimited by the cell wall was the main factor associated with retention or passage. The filters containing the giant cells were incubated in PBS with shaking for 20 minutes, and the cells were concentrated by centrifugation. After filtration, we observed that the population was significantly enriched in giant cells, being more than 90% of the sample. Finally, the cells that transited or were retained by the filter were counted using a haemocytometer.

Once regular and giant cells were obtained and exposed to the macrophages, the 96-wells plate was placed under a Leica DMI 4000B microscope using a 20× objective with a 5% CO_2_ environment and 37°C. Pictures were taken at different time intervals (see figure legend of the corresponding supporting videos). The videos generated by the Leica software were exported as .avi documents and processed with ImageJ (National Institutes of Health, USA, http://rsb.info.nih.gov/ij/index.html) and VidCrop 2.1.0.0 (GeoVid) softwares. The final videos were generated by merging 5 frames per second.

### Melanin detection by immunofluorescence

Suspensions of cells isolated from mouse lung were air-dried on poly-L-lysine-coated slides (Sigma). The slides coated with the cells were washed in PBS, incubated in blocking buffer (Pierce, Rockford, IL) for 1 h at 37°C followed by incubation with 10 µg/ml of the IgM melanin-binding monoclonal antibody (mAb) 6D2 for 1 h at 37°C. MAb 6D2 was generated against melanin derived from *C. neoformans* [27]. After washing, the slides were incubated with a 1∶1000 dilution of tetramethyl rhodamine isothiocyanate (TRITC) -conjugated goat anti-mouse (GAM) IgM (Southern Biotechnologies Associates, Inc; Birmingham, AL) for 1 h at 37°C. The slides were washed, mounted using a 50% glycerol, 50% PBS, and 0.1 M N-propyl gallate solution, and viewed with an Olympus AX70 microscope equipped with fluorescent filters. Negative controls consisted of cells incubated with mAb 5C11, which binds mycobacterial lipoarabinomannan [65], as the primary Ab or with TRITC-labeled Ab alone.

### Scanning electron microscopy

Yeast cells were washed in PBS and suspended in fixing solution (2% p-formaldahyde, 2.5% glutaraldehyde, 0.1 M sodium cacolydate). Cells were then serially dehydrated with ethanol, coated with gold palladium and visualized using a JEOL (Tokyo, Japan) JAM 6400 microscope.

### Transmission electron microscopy

Cells grown in vitro or isolated from the lungs of infected mice (see above) were fixed with 2.5% glutaraldehyde in 0.1 M sodium cacodylate buffer. The cells were treated with osmium tetraoxide and serially dehydrated. The samples were embedded in epoxy resin and ultrathin sections were obtained, stained with uranyl acetate and lead citrate, and observed in a CM12-Phillips transmission electron microscope.

### DMSO and γ-radiation treatment

Yeast cells with enlarged capsule were exposed to varying amounts of γ-radiation from ^137^Cs to remove layers of the polysaccharide capsule as described [29], [66]. Briefly, giant and in vitro-grown cells were washed three times in PBS to remove shed capsular polysaccharides, suspended in 1 mL of distilled H_2_O, and irradiated using a Shepherd Mark I Irradiator at the dose rate of 1388 rads/min. For all experiments, cells were irradiated for 40 minutes. Irradiated cells were collected by centrifugation. In other experiments, the fungal cells were suspended in DMSO as described in [29]. The presence of capsule after the treatments (γ-irradiation or DMSO) was visually observed by suspending the cells in India Ink and regular microscopy.

### Complement labelling and detection

Complement (C3; complement protein 3) deposition on the cryptococcal capsule was performed as in [20]. Briefly, C57BL/6J mice were bled from the retro-orbital cavity and serum was obtained by centrifugation. Approximately 2×10^7^ cryptococcal cells were suspended in 700 µL freshly-obtained serum, and incubated at 37°C for 1 h. Cells were extensively washed and suspended in PBS. C3 was then detected using a fluorescein-isothiocyanate (FITC) conjugated GAM C3 antibody (4 µg/mL, Cappel, ICN, Aurora, OH). Yeast washed and not suspended in serum were used as controls. To delineate the capsular edge, mAb 18B7 (10 µg/mL) specific for GXM [67] was added, and detected using a TRITC conjugated GAM IgG1 antibody (10 µg/ml, Southern Biotechnology Associates, Inc). The cells were observed under fluorescent filters with the Olympus AX70 microscope, QCapture Suite V2.46 software for Windows, and Adobe Photoshop 7.0 for Macintosh.

### Imaging of daughter cell emergence from the giant mother cells

Yeast cells were isolated from infected mice as described above, and placed on Sabouraud agar plates for 18 h 30°C. Initially, we tried recording the budding of giant cells by basic microscopy techniques, such as taking pictures every few minutes or seconds. However, the separation of the bud through the capsule of the giant cell was too fast, so we developed a new approach to record the phenomenon. The surface agar plate was observed with an Olympus AX70 microscope to visualize and continuously record giant cells. To record real-time daughter cell emergence, the cells were observed in the computer screen with the “Preview” option, and the image of the screen was recorded with a Digital Handycam Sony Camcorder affixed to a tripod. The videos were converted into digital files using Windows Movie Maker software provided by Microsoft Windows and processed with the Quick Media Converter (V. 3.6.5) software. Although this method provided lower resolution than the regular CCD used in microscopy, it permitted a precise measurement of the phenomenon.

### XTT viability assay

Giant cells were obtained by filtering the yeasts obtained from the lung of infected mice through 22 µm filters. Then, the yeast cells were separated from the filter by gently shaking the filters in 20 mL of water in 50 mL centrifuge tubes. After 20 minutes, the filters were removed, and the tubes centrifuged at 2000 r.p.m. Then, the cells were suspended in 2 mL of sterile water and the cell concentration was estimated using a haemocytometer. Approximately 10^5^ giant cells were placed on 96 wells plates. In parallel, regular cells were obtained by overnight incubation in Sabouraud, washed with sterile water and counted with a haemocytometer. Then, the same number of cells (10^5^) was placed in 96-wells plates. As negative controls, equal numbers of giant and regular cells were heat-inactivated (45 minutes at 60°C) and placed in different wells of the 96-wells plates. Viability measurement based on the reduction of 2,3-bis(2-methoxy-4-nitro-5-sulfophenyl)-2H-tetrazolium-5-carboxanilide inner salt (XTT) by living cells was performed as described in [21] with minor modifications, which involved the use of 1 mg/mL of XTT and 25 µM menadione. Optical density at 450 nm was recorded every 30 minutes for 18 hours in a iEMS Spectrophotometer (Thermofisher). Differences in metabolic activities were calculated by fold differences in the optical densities of the different wells.

### Indirect immunofluorescence

To detect capsular features, we observed the immunofluorescence pattern after incubating the cells with the mAb 18B7 to the capsular polysaccharide as described above (see complement labelling and detection section), but using goat anti-mouse IgG1-FITC conjugated as the detection Ab. Yeast washed and incubated with the IgG1-FITC alone were used as controls. In some experiments, calcofluor (10 µg/mL) was included to visualize the cell wall.

### Wheat germ agglutinin staining

To observe the presence of chitin-like structures, fungal cells with enlarged capsule (incubated in 10% Sabouradud in 50 mM MOPS buffer pH 7.3) were treated as in [33]. Briefly, the cells were washed with PBS and suspended in 4% p-formaldehyde cacodylate buffer (0.1 M, pH 7.2) and incubated for 30 min at room temperature. The fixed cells were washed in PBS and suspended in 100 µl of a 5 µg/mL of WGA conjugated to Alexa 594 (Molecular Probes, Invitrogen) for 1 hour at 37°C. Cell suspensions were mounted over glass slides and photographed with a Leica DMI 3000B fluorescence microscope.

### Vacuole staining and visualization by confocal microscopy

To identify the vacuole in the yeast cells, we used the specific dye MDY-64 (Molecular Probes, Invitrogen, Eugene, Oregon) following the manufacturer's recommendations. Briefly, the cells were suspended in 10 mM HEPES buffer (pH 7.4) supplemented with 5% glucose. MDY-64 was dissolved in DMSO, and added to 10^6^ cells at a final concentration of 10 µM. The cells were incubated for 5 minutes at room temperature, and washed twice with the same buffer. The cells were observed with a SP5 confocal microscope (Leica Microsystems).

### Cellular DNA content

Cells were isolated as described above and fixed by heating the cells at 60°C for 45 minutes in PBS buffer. Then, the cells were separated in two parallel samples, and propidium iodide was added to one of them at a final concentration of 10 µg/mL. DNA content was analyzed using a FACSCalibur cytometer (Becton Dickinson). As a control, cells grown in vitro in Sabouraud medium were also analyzed.

### Nuclear staining

To visualize the nucleus, the cells were treated with 3.7% formaldehyde for 30 minutes. Then, the cells were washed with PBS and DAPI was added at 0.3 µg/mL. The cells were incubated for 10 minutes at 37°C, and then washed twice with PBS. Finally, fluorescence was visualized in a Leica DM3000 microscope.

### Real-time PCR

Giant cells were obtained by filtering the lung extracts through 22 µm filters as above. The filters were then placed in 50 mL tubes containing 20 mL of sterile water with moderate shaking, and after 20 minutes, the filters were removed. The tubes were centrifuged, and the pellet suspended in 0.5 mL of sterile water. Then, the cell concentration was determined using a haemocytometer. In parallel, cells obtained from a fresh liquid culture in Sabouraud were counted, and a cell suspension was prepared at the same concentration as that calculated for the giant cells. A real-time PCR using whole cells was then designed using equivalent numbers of the different cell types in the well. The reaction (final volume of 20 µL) contained 2.8×10^3^ or 2.8×10^2^ cells, 1.5 mM MgCl_2_, and 0.8 µM of ITS1 (5′TCCGTAGGTGAACCTGCGG3′) and ITS2 (5′GCTGCGTTCTTCATCGATGC3′) oligonucleotides, which amplify the ITS1 region from the ribosomal DNA. The real time was performed using the SensiMix Kit (Quantance) using the enzymes and SYBR green concentrations recommended by the manufacturer. The reaction mix was placed in a 96-wells plate and PCR was performed in a LC480 real-time PCR machine (Roche). We included wells with a known concentration of *C. neoformans* genomic DNA (20, 2, 0.2 and 0.02 ng) to quantify the results. The PCR was performed according to the following protocol: initial step of 10 minutes at 95°C and 45 amplification cycles (10 seconds at 95°C, 5 seconds at 54°C and 30 seconds at 72°C). Once the PCR was finished, a standard curve was calculated using the wells of the known genomic DNA concentration, and this curve was used to calculate the estimate of DNA present in each of the samples.

### Classification and Regression Trees (CART) analysis

The CART system was proposed by Breiman et al. [68], and is characterized by binary-split searches, automatic self-validation procedures and surrogate splitters. This analysis is used to find associations between events with statistical support. CART analysis (CART 6.0 Salford Systems, Ca., USA) was used to find associations between giant cell formation and inflammation in the lungs. This analysis was performed with the following methodological conditions, Gini method, minimum cost tree regardless of the size for selecting the best tree, 10 v-fold-cross-validation, equal priors, no costs, and no penalties. Relative error of 0 means no error or perfect fit, whereas 1 represents the performance of random guessing. The statistical support for this association is given by the ROC curve. In this graph, specificity (false positive rate) vs. sensitivity (true positives rate) is calculated, and the area under the curve is analysed. When this area is 1 (100% of sensitivity and 0% false positives), a total agreement for the prediction is obtained. An area of 0.5 or below is indicative of random guess.

### Oxidative stress susceptibility

Yeast cells (regular and giant) were obtained as described above. The cells were incubated in PBS with or without 1 mM H_2_O_2_ at a cell density of 10^4^ cells/mL. After two hours of incubation at 37°C, 100 µL of each sample was plated on Sabouraud agar medium. In addition, a 1/10 dilution was done in PBS, and 100 µL of this dilution was also plated. The plates were incubated at 30°C for 48 hours and the colonies were enumerated. The survival was expressed as the percentage of colonies counted in the samples incubated with H_2_O_2_ compared to colonies of control samples not exposed to the oxidative agent.

### Statistical analysis

Normal distribution in group samples were assessed using the Shapiro-Wilk and Kolmogorov-Smirnov tests using Unistat 5.0 (Unistat Ltd, London, England) and Analyse-it (Analyse-it Ltd, Leeds, England) softwares for Excel. Statistical differences between groups were tested using Student's t-Test (normal distributions) or Kruskal-Wallis test (non-parametric test for non-normally distributed samples). Differences were considered significant when *p* value was below 0.05.

### Ethics statement

All the experiments involving the use of animals have been performed following the guidelines of the Bioethical and Animal Welfare Committee of the Instituto de Salud Carlos III (approved protocol PA-349, to be performed at the National Centre for Microbiology).

## Supporting Information

Video S1 Live imaging of giant cells budding in vitro. Giant cells were obtained from infected mice as described in Materials and Methods and in Figure 4 legend. Live imaging of budding was recorded and processed as described in Materials and Methods.
(2.39 MB AVI)

Video S2 Live imaging of giant cells budding in vitro. Giant cells were obtained from infected mice as described in Materials and Methods and in Figure 4 legend. Live imaging of budding was recorded and processed as described in Materials and Methods.
(0.18 MB WMV)

Video S3 Live imaging of giant cells budding in vitro. Giant cells were obtained from infected mice as described in Materials and Methods and in Figure 4 legend. Live imaging of budding was recorded and processed as described in Materials and Methods.
(1.71 MB AVI)

Video S4 Live imaging of giant cells budding in vitro. Giant cells were obtained from infected mice as described in Materials and Methods and in Figure 4 legend. Live imaging of budding was recorded and processed as described in Materials and Methods.
(2.94 MB AVI)

Video S5 Phagocytosis of *C. neoformans* by murine-like macrophages. The video shows the interaction of RAW264.7 macrophage cell lines exposed to *C. neoformans* H99 strain at a ratio 1∶2. Videos were performed as described in Materials and Methods. Pictures were taken every 5 minutes, and 5 frames per second are shown in the video (1 second of the video is equivalent to 25 minutes of real time). A field of 100 µm width is shown.
(5.46 MB AVI)

Video S6 Macrophage fusion after *C. neoformans* phagocytosis and cell division. RAW264.7 macrophage cell lines were exposed to *C. neoformans* H99 strain at a ratio 1∶2. Videos were performed as described in Materials and Methods. Pictures were taken every 5 minutes, and 5 frames per second are shown in the video (1 second of the video is equivalent to 25 minutes of real time). A field of 100 µm width is shown.
(6.16 MB AVI)

Video S7 *C. neoformans* intracellular replication. Macrophages and *C. neoformans* grown in Sabouraud were mixed as described in supplemental Videos S5 and S6. The pictures were taken every 2 minutes, and 5 frames per seconds are shown in the video (1 second of the video is equivalent to 10 minutes of real time).
(6.16 MB AVI)

Video S8 Interaction between giant cells and macrophages. Macrophages and *C. neoformans* giant cells were mixed at 1∶2 ratio as described in Materials and Methods. Pictures were taken every 3 minutes, and 5 frames per second are shown in the video (1 second of the video is equivalent to 15 minutes of real time). A field of 100 µm width is shown.
(5.09 MB AVI)

Video S9 Interaction between giant cells and macrophages. Macrophages and *C. neoformans* giant cells were mixed at 1∶2 ratio as described in Materials and Methods. Pictures and video were taken and assemble as described in Supporting Video S8. Scale bar denotes 50 µm.
(2.04 MB WMV)
