# Correction: Fungal Cell Gigantism during Mammalian Infection

**DOI:** 10.1371/annotation/0675044c-d80f-456f-bb63-4f85fb1d0c33

**Published:** 2010-06-23

**Authors:** Oscar Zaragoza, Rocío García-Rodas, Joshua D. Nosanchuk, Manuel Cuenca-Estrella, Juan Luis Rodríguez-Tudela, Arturo Casadevall

The publisher wishes to note that the "Accept" date on this article reflects the "Production Accept" date. This article was accepted by the editor on 16 April 2010, while its companion article (Okagaki LH, Strain AK, Nielsen JN, Charlier C, Baltes NJ, et al. (2010) Cryptococcal Cell Morphology Affects Host Cell Interactions and Pathogenicity. PLoS Pathog 6(6): e1000953) was accepted by the editor on 27 January 2010. 

